# Multifunctional Polymeric Nanoplatforms for Brain Diseases Diagnosis, Therapy and Theranostics

**DOI:** 10.3390/biomedicines8010013

**Published:** 2020-01-13

**Authors:** Shahryar Shakeri, Milad Ashrafizadeh, Ali Zarrabi, Rasoul Roghanian, Elham Ghasemipour Afshar, Abbas Pardakhty, Reza Mohammadinejad, Anuj Kumar, Vijay Kumar Thakur

**Affiliations:** 1 Department of Biotechnology, Institute of Science and High Technology and Environmental Sciences, Graduate University of Advanced Technology, Kerman 7631818356, Iran; sh.shakeri@kgut.ac.ir; 2 Department of Basic Science, Faculty of Veterinary Medicine, University of Tabriz, Tabriz 5166616471, Iran; dvm.milad73@yahoo.com; 3 Sabanci University Nanotechnology Research and Application Center (SUNUM), Tuzla 34956, Istanbul, Turkey; alizarrabi@sabanciuniv.edu; 4 Department of Biology, Faculty of Sciences, University of Isfahan, Isfahan 81746, Iran; r.roghanian@sci.ui.ac.ir; 5 Neuroscience Research Center, Institute of Neuropharmacology, Kerman University of Medical Sciences, Kerman 7619813159, Iran; elham_gh_afshar@yahoo.com; 6 Pharmaceutics Research Center, Institute of Neuropharmacology, Kerman University of Medical Sciences, Kerman 7616911319, Iran; drpardakhti@yahoo.com; 7 School of Chemical Engineering, Yeungnam University, 280 Daehak-ro, Gyeongsan 38541, Korea; 8 Enhanced Composites and Structures Center, School of Aerospace, Transport and Manufacturing, Cranfield University, Bedfordshire MK43 0AL, UK

**Keywords:** blood–brain barrier (BBB), central nervous system (CNS), nanotechnology, drug delivery systems, polymeric nanoparticles, theranostics

## Abstract

The blood–brain barrier (BBB) acts as a barrier to prevent the central nervous system (CNS) from damage by substances that originate from the blood circulation. The BBB limits drug penetration into the brain and is one of the major clinical obstacles to the treatment of CNS diseases. Nanotechnology-based delivery systems have been tested for overcoming this barrier and releasing related drugs into the brain matrix. In this review, nanoparticles (NPs) from simple to developed delivery systems are discussed for the delivery of a drug to the brain. This review particularly focuses on polymeric nanomaterials that have been used for CNS treatment. Polymeric NPs such as polylactide (PLA), poly (D, L-lactide-co-glycolide) (PLGA), poly (ε-caprolactone) (PCL), poly (alkyl cyanoacrylate) (PACA), human serum albumin (HSA), gelatin, and chitosan are discussed in detail.

## 1. Introduction

Central nervous system (CNS) disorders are among the most common and complex diseases known in humans [1,2]. The best known brain diseases include cancers, neurodegenerative disorders, HIV encephalopathy, and inflammatory diseases [3,4]. Most cancer-related deaths in patients below the age of 35 have been reported as arising from brain cancer [5,6,7]. The different kinds of brain tumors are neuroepithelial, meningeal, primary CNS lymphomas, tumors of the sellar region, and metastatic [8,9,10]. Alzheimer’s (AD), Parkinson’s (PD), multiple sclerosis (MS), Huntington’s, and prion diseases are the main neurodegenerative diseases (NDDs) that affect the neuron cells in a detrimental way [11,12,13,14]. NDDs lead to the loss or death of neural cell function and have symptoms related to movement, memory, and dementia. There are some unsolved problems in the clinic about the treatment of brain diseases [15,16,17]. In the past decade, CNS disorders represented one of the largest markets for the development of new treatments. This market reached US$75.3 billion in 2010 and $102.0 billion in 2015 [18,19,20,21]. Fortune Business Insights, in a recent report entitled “The Neurodegenerative Diseases Drugs Market: Global Market Analysis, Insights and Forecast, 2019–2026,” gives valuable predictions about the market. In 2018, the global market value was $35,497.3 million and is expected to grow to $62,786.2 million by 2026 [22].

## 2. The Blood–Brain Barrier (BBB)

The blood–brain barrier (BBB) has a special structure that separates the extracellular fluid of neurons from blood circulation [23,24,25,26,27,28,29]. Paul Ehrlich gave the first evidence for the presence of BBB in his research in 1885 [30]. The BBB functions as a barrier for the complete separation of blood circulation from the fluid inside of the CNS and protects nerve cells from damage by foreign substances and infections that originate from the blood [31]. In addition, the BBB prevents water-soluble molecules, proteins, peptides, genes, and antibiotics with a molecular weight of above 500Da from reaching the brain, although NPs of such molecular weights could pass through it owing to their aspect ratio and spatial geometry [32,33]. This special barrier is composed of different kinds of cells. Endothelial cells, pericytes, astrocytes, and microglia are incorporated in the 3D structure of the BBB [34]. Endothelial cells in the structure of BBB have different characteristics from their counterparts in the periphery, including a high content of mitochondrial cells and changed pinocytic activity. The tight junctions between the neighboring endothelial cells are complex and formed by several transmembrane proteins. Pericytes are located at the inner brain membrane and are covered by basal lamina and proteins [35,36]. Astrocytes and their endfeet have been attached to the walls of capillaries and help steady the capillary structure [37]. The BBB is the main problem in the treatment of CNS diseases. Therefore, overcoming this barrier is the most critical area of research for CNS disease therapy [38,39].

There are two current strategies: paracellular and transcellular transport for passive or active crossing of the BBB, respectively [40]. Temporary disruption of the BBB, transcytosis, and nanoplatforms facilitate the delivery of molecules into the CNS [41]. Molecules use active efflux transporters, carrier-mediated transporters, and receptor-mediated transporters to cross the BBB. Nanoconjugation of ligands targeting endothelial cell surface receptors facilitates the endocytosis of nanocarriers. Iron-transferrin, insulin, metabolic nutrient transporters, low-density lipoprotein (LDL) cholesterol, nicotinic acetylcholine (nAchR), and integrin are the most explored receptors for transporting drugs across the BBB [42].

## 3. Nano-Scale Drug Delivery to the Brain

In recent years, a wide variety of studies have been performed in the field of drug delivery into the brain [43,44,45,46]. However, the BBB is a barrier to the successful delivery of drugs to the CNS since only a small number of molecules can cross the BBB [47]. Almost all (>98%) small molecules can cross the BBB, whereas high molecular weight drugs such as proteins, peptides, monoclonal antibodies, and genes are not able to penetrate through the BBB and access the CNS [48,49,50]. Hence, novel technologies and delivery systems are needed to overcome this barrier and release the drugs into the brain matrix. Nanotechnology-based drug delivery systems are a powerful method for drug transport into the brain [40,51,52]. A well-known candidate for CNS targeted delivery of drugs is colloidal-based particulate systems [53,54,55]. NPs are used in the form of nanospheres as well as nanocapsules, and the drug is entrapped inside the matrix or attached to the surface [56,57]. Nanomaterials penetrate small capillaries because of their size; cells absorb them and the drug will be released into their compartment or cytoplasm. Sustained drug release at the targeted site is one of the advantages of NPs that are prepared with biodegradable materials [58,59,60]. NPs made of polymeric materials are solid particles ranging in size from 1 to 100 nm in at least one dimension. They are nontoxic, nonimmunogenic, and stable in the blood if they are appropriately surface engineered with proper ligands [61,62]. The drugs stay inside or at the surface of biodegradable NPs and will be released in a tunable manner at a specific site in the body. In this way, the targeted delivery of drugs will enhance therapeutic efficiency and reduce drug toxicity and side effects [42,63]. Reduction of adverse side effects and improvements in the bioavailability of drugs with a short half-life are other potential benefits of NPs-based drug-delivery systems [64,65]. Nanotechnology can be applied for the delivery of drugs to the brain for the treatment of brain-related diseases. For instance, several drugs such as growth factors and neuropeptides cannot cross the BBB and so are ineffective when administered intravenously (IV) [66,67]. Therefore, numerous efforts have been focused on the use of NPs for the delivery of drugs into the brain [18].

### Development of the Nanoparticulate Systems: From Simple to Targeted Generations

Long-time circulation in the bloodstream is one of the most important characteristics of NPs after IV administration [68,69]. The reticuloendothelial system (RES) is an active agent in the removal of the first generation of NPs (without the capability of escape from RES) from the blood circulation. Some characteristics of NPs such as size and surface charge help them to escape from clearance property of RES and endure more time in circulation [70]. Moreover, the particles’ size and charge could affect their cellular uptake and cytotoxicity, so that particles with a size of less than 500 nm and a positive charge are more suitable for cell uptake. In other words, particles ˂ 200 nm are preferred for systemic applications. It is important to remark that particles smaller than 5 nm are also not suitable since they may be removed from the circulation by renal excretion. Moreover, they are hazardous since they can directly affect the important biological macromolecules of the nucleus (DNA and RNA). On the other hand, positively charged particles also facilitate interactions with negatively charged cell membranes, along with increasing the cytotoxicity. These positively charged particles can also improve the interaction of NPs with other types of biological components, such as proteins, that restrict their circulation time, while particles with a neutral or negative charge have longer circulation times [71,72,73].

The surface modification of NPs with different molecules such as polyethylene glycol (PEG) is a promising strategy for increasing the half-life and persistence in the bloodstream (second generation of NPs). An external surface layer of hydrophilic PEG chains around the hydrophobic polymeric matrix camouflages particles from recognition by RES and provides a long plasma residence time [74]. In addition to the long-time circulation, the good affinity of NPs for the targeted tissue is another important factor in drug delivery [75]. It has been shown that PEGylated NPs have a low affinity to the brain [74]. The first attempt at the delivery of the drug to the brain was made by Kreuter et al. [76]. They used polysorbate 80-coated poly (butyl cyanoacrylate) (PBCA) NPs for the delivery of dalargin into the CNS (third generation of NPs). This kind of NP has a good affinity to the brain in addition to long-time circulation, and several drugs have been successfully delivered into the brain by this system [77,78,79]. Various types of nanosystems have been evaluated for drug delivery to the brain [80,81,82]. Receptor-mediated transporter systems are modern examples of these NPs [83,84,85]. The effective delivery of drugs to the specific target site (e.g., the brain) is achieved by the ligand–receptor mechanism. The presence of ligands on the surface of drug-loaded NPs can deliver the carrier system to the target sites with specific receptors [41,86,87,88,89]. For instance, some ligands such as transferrin (Tf), Apolipoprotein (Apo) E, B, A, and antibodies (Tf receptor or OX26) on the surface of NPs have been shown to pass through the BBB and allow targeted delivery of carriers into the brain parenchyma via receptor-mediated endocytosis [90,91,92,93,94,95,96]. For example, PEGylated albumin or chitosan (CS) NPs coupled with OX-26 were proposed as a potential candidate for targeted brain delivery [97,98]. The main mechanism for the delivery of these NPs through the BBB is receptor-mediated transport endocytosis.

## 4. Polymeric Nanoparticles for Brain Disease Theranostics

**Figure 1:**
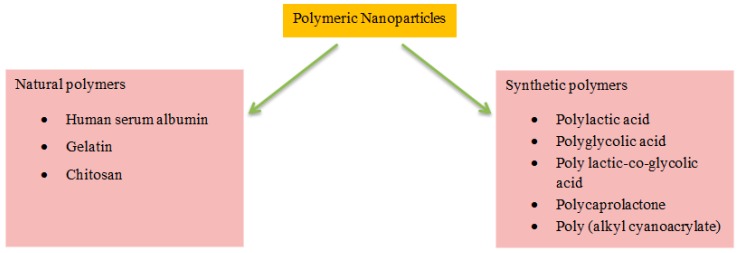
Selected polymeric NPs for the delivery of drugs to the brain.

The therapeutic benefits of various water-soluble/insoluble drugs and bioactive agents, such as solubility, bioavailability, and retention time, are promoted by the frequent use of biodegradable polymer-based NPs [99,100]. A wide range of polymeric biomaterials are used as a matrix for drug delivery nanosystems [101]. Polymeric biomaterials include synthetic and natural ones, and their hybrid polymers. For the successful application of these polymeric systems in medicine and pharmaceuticals, one must consider the biocompatibility, biodegradability, and nontoxic, nonimmunogenic, and noncarcinogenic characteristics of these materials [102]. Herein, we have discussed synthetic and natural polymers for application in the field of nanotechnology-based delivery systems to the brain (Figure 1) (Table 1).

### 4.1. Synthetic Polymers

#### 4.1.1. Polyesters

Synthetic polymers have been widely used for the delivery of bioactive agents and drugs [44,163,164,165,166,167,168,169,170,171,172]. Among them, polyesters have great potential because of their biocompatibility and biodegradability. They are toxicologically safe and their monomers, as well as by-products, are eliminated by the metabolic pathways of the human body [173]. Polylactic acid (PLA), polyglycolic acid (PGA), and PLGA have been widely used in medicine and pharmaceuticals [174]. PLA and PLGA have been approved by the FDA for clinical uses [112]. These polymers degrade in the human body without any induction of inflammation or immune reactions [175]. They have been applied for the fabrication of biodegradable medical devices such as scaffolds and drug-loaded NPs or implants [176].

#### 4.1.2. PLA

**Figure 2:**
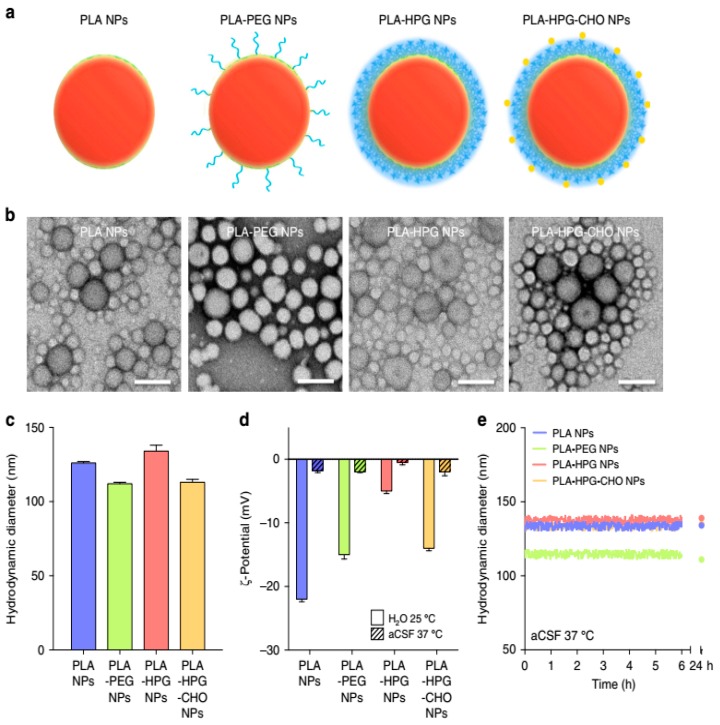
(**a**) Schematic representation of PLA-based NPs having different coating materials. (**b**) Population and morphology of NPs using TEM (scale bar = 100 nm). (**c**) Characterization of NPs for hydrodynamic diameters using dynamic light scattering (**d**), zeta potential using laser doppler anemometry (**e**), and analysis of particle size (in CSF at 37 °C) is observed as stable and no measurable aggregation was observed up to 24 h. Reproduced with permission from Song, E.; Gaudin, A.; King, A.R.; Seo, Y.E.; Suh, H.W.; Deng, Y.; Cui, J.; Tietjen, G.T.; Huttner, A.; Saltzman, W.M. Surface chemistry governs cellular tropism of nanoparticles in the brain. *Nat. Commun.* 2017 [108].

PLA is a biocompatible polymer and biodegradable in the human body that degrades into its monomeric units [177]. The monomer of lactic acid is a safe and natural intermediate in carbohydrate metabolism. Cheng et al. [103] studied the delivery of neurotoxin-I (NT-I) using PLA NPs. The authors used PLA instead of PBCA to prevent the toxicity of the by-products. The level of NT-I was found to increase in the brain after IN or intravascular (IV) administration of NT-I–PLA NPs. Results have demonstrated that IN administration of NT-I–PLA NPs was more effective than intravascular administration. Kubek et al. [105] used thyrotropin-releasing hormone (TRH) for loading into the PLA NPs for the IN administration of seizure. Although the results were not as expected, the authors believe that IN administration of biodegradable NPs can be effective in the treatment of seizures. Hu et al. [178] studied the use of lactoferrin (Lf)-conjugated PEG–PLA NPs to deliver a fluorescent dye (coumarin-6) into the mouse brain. An IV injection of Lf-PEG–PLA NPs demonstrated that the NPs’ entrance into the brain was increased 3-fold by coumarin-6 in the mouse brain compared to NPs without Lf. The authors suggested that the Lf-PEG–PLA NPs system can provide a novel system for brain drug delivery, especially targeting peptides, proteins, and genes. Tf was another ligand used by Gan and Feng [106]. They fabricated Tf-conjugated NPs of PLA-d-α-Tocopheryl polyethylene glycol succinate (PLA-TPGS) diblock copolymer for the delivery of imaging and therapeutic agents. The results showed the higher effectiveness of Tf-decorated NPs for the delivery of agents across the BBB. Cell-penetrating peptides (CPPs) with a low content of basic amino acids were reported as good candidates for the functionalization of PEG–PLA NPs and drug delivery to the brain [107]. Increased accumulation of functionalized NPs was observed in the brain. Song et al. prepared polylactic acid NPs to evaluate their efficiency in brain delivery (Figure 2) [108]. They have shown that the surface properties of NPs were associated with their cellular distribution, and used them in in vitro and in vivo studies to test the idea. Overall, it was concluded that PLA NPs are potential candidates for the delivery of therapeutics into the brain due to their low toxicity and high uptake by brain cells.

Zheng et al. designed H102-loaded PEG-PLG NPs for efficient delivery into the brain in the case of AD [109]. In this case, some NPs are able to cross the BBB and are taken up by caveolae-mediated endocytosis. Interestingly, H102-loaded PEG-PLG NPs have shown excellent biocompatibility and, simultaneously, good therapeutic efficiency in reducing Aβ plaques, enhancing Aβ-degrading enzymes, decreasing tau protein phosphorylation, protecting synapses, and promoting spatial learning and memory. Pan et al. investigated the delivery of α-asarone into the brain by lactoferrin-modified mPEG–PLA NPs [110]. They prepared NPs using premix membrane emulsification and used IN administration. These NPs efficiently delivered α-asarone into the brain and displayed good permeability and bioavailability. Interestingly, it was found that lactoferrin moiety is involved in increasing the efficacy of brain targeting, reducing liver accumulation, and reducing the level of toxicity on nasal mucosal cilia and epithelial cells. Shen et al. prepared low-density lipoprotein receptor (LDLR) peptide-conjugated polylactic acid (PLA)-coated mesoporous silica NPs for the delivery of resveratrol into the brain [111]. PLA coating was used as an occlusion for resveratrol burst release and they also used reactive oxygen species (ROS) to facilitate PLA degradation and induce drug release. It was found that LDLR ligand peptides increase the migration of NPs through the BBB and remarkably decrease the stimulation of microglial cells by phorbol myristate acetate or lipopolysaccharide, leading to the efficiency of these NPs in treating oxidative stress in the CNS.

Wang and co-workers synthesized cationic lipid assisted PEG–PLA NPs to prevent microglial neurotoxicity [179]. They prepared NPs using a double-emulsion solvent evaporation technique and then loaded complement component C3-siRNA on NPs to inhibit microglial neurotoxicity after cerebral ischemia/reperfusion (I/R) injury. It was found that these NPs potentially penetrate the BBB and remarkably reduce the expression of C3 in microglial cells as well as simultaneously decrease the number of inflammatory cells and pro-inflammatory factors in the penumbra, resulting in efficient improvement of the brain I/R injury. Zhu et al. designed tumor-specific protease-activated cell-penetrating peptide (ACPP)-conjugated micelles for treating brain gliomas [180]. In vitro and in vivo studies demonstrated good uptake and intracellular drug release of micelles. Also, these micelles were found to efficiently penetrate the BBB and, using ACPP, promoted the survival of mice bearing gliomas. Furthermore, these micelles had lower toxicity.

#### 4.1.3. PLGA

Various studies have been performed to fabricate PLGA NPs and scaffolds [181]. The biodegradability, biocompatibility, and long-lasting and sustained release properties of PLGA make it a suitable polymer for biomedical and pharmaceutical applications [178,182]. The polymer degradation and drug-releasing profile can be affected by changes in molecular weight and the molar ratio of lactic acid to glycolic acid [112]. Both monomers are consumed and eliminated during the normal metabolism of the cells [183]. Biodegradable delivery systems based on the PLGA polymer have been used in the imaging, diagnostics, and treatment of diseases [184,185,186,187]. Entrapment of various types of drugs such as proteins, peptides, genes, and anticancer drugs has been performed in PLGA NPs [188,189,190,191]. Protein and peptide drugs are susceptible to high temperature or acidic environments. Long-term exposure of proteins and peptides to the acidic by-products of PLGA can decrease the stability and bioavailability after polymer degradation [192]. So, it is important to determine the physicochemical characteristics of proteins and peptides.

**Figure 3:**
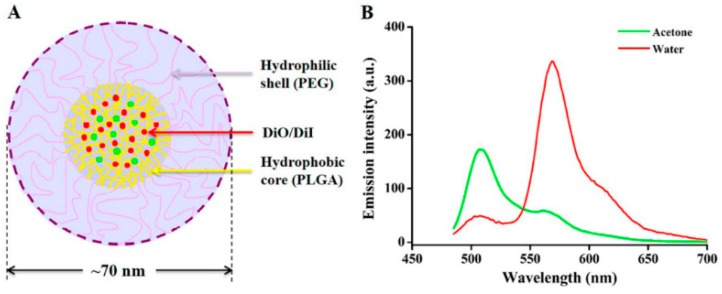
(**A**) Schematic diagram of DiO/DiI (1:1) NPs with each at 0.5% weight ratio of the polymer and (**B**) fluorescence spectra of DiO/DiI NPs with 10-fold dilution in water (red) and acetone (green). Reproduced with permission from Chen, T.; Li, C.; Li, Y.; Yi, X.; Wang, R.; Lee, S.M.Y.; Zheng, Y. Small-sized mPEG–PLGA nanoparticles of Schisantherin A with sustained release for enhanced brain uptake and anti-parkinsonian activity. *ACS Appl. Mater. Interfaces* 2017 [118].

PLGA NPs have been investigated for the treatment of brain diseases. Tahara et al. [112] studied different surface-modified PLGA NPs for delivery to the brain. The authors used CS, polysorbate 80 (P80), and poloxamer 188 (P188) as surface modifier agents in their studies. NPs were prepared by the emulsion solvent diffusion method. After carotid artery injection, P80-PLGA NPs were found to exhibit prolonged circulation in the blood compared to the other NPs, and their concentration in the brain was increased. In addition, the cellular uptake of CS-PLGA NPs was higher due to electrostatic interaction with the cell membrane. Budhian et al. [193] showed that hydroxyl-terminated PLGA NPs can release haloperidol over a long period as compared to methyl-terminated PLGA NPs. Haloperidol is an antipsychotic drug used for schizophrenia therapy. Gelperina et al. [113] have used surfactant-coated PLGA NPs for the delivery of DOX and loperamide to the brain. In this study, polyvinyl alcohol (PVA) and human serum albumin (HSA) were used as stabilizers, while P80 and P188 were used as coating surfactants for the formulation of PLGA NPs. Results showed that DOX-PLGA/PVA+P188 NPs were most effective and had a high antitumor effect. DOX-PLGA/HSA+P188 NPs also exhibited a high antitumor effect and produced long-term remission in the tested animals. The effect of Lop-PLGA/PVA+P80 and Lop-PLGA/HSA+P188 NPs was also considerable. The effect of the surfactants on the efficiency of the HSA-stabilized particles was less than that of particles stabilized by PVA. The high antitumor effect against glioblastomas, as well as considerable analgesia, revealed that these NP systems can cross the BBB and release the drug at a specific site. Similar results were also observed by Chen et al. [118]. Block copolymers were also used for the preparation of NPs [194]. Loperamide-loaded PLGA-PEG-PLGA NPs with a surface modified by poloxamer 188 or polysorbate 80 were used for in vitro BBB penetration. Mittal et al. [114] studied Tween 80-coated PLGA NPs for the delivery of estradiol to the brain upon oral administration in a rat model of AD. Results showed that a high level of estradiol was detectable in the brain after oral administration. In addition, the suppression of Aβ42 expression by estradiol showed that Tween 80–PLGA NPs could deliver estradiol to the brain by the oral administration route. Surface modification of PLGA NPs with TMC was carried out by Wang et al. [115]. Coenzyme Q10-loaded TMC/PLGA NPs were found to improve memory impairment after injection. In addition, the NPs were found to exhibit low toxicity and good penetration into the brain matrix. Pep TGN, a novel 12 amino acid peptide, was used as a ligand that was attached to the surfaces of PEG-PLGA NPs for targeting the brain. High accumulation of NPs was observed in the brain after IV injection [123]. Work was done by other researchers to prepare and fabricate NPs using PLGA polymers. These polymeric NPs can target brain cancer and other diseases [195,196]. Sanchez-Lopez et al. developed PLGA PEGylated NPs for the delivery of memantine in AD [117]. MTT tests showed that these NPs are safe for brain cell lines (bEnd.3 and astrocytes) and PLGA PEGylated NPs could penetrate the BBB. It was found that these NPs containing memantine decrease Aβ plaques and related inflammation characteristics of AD. Chen et al. designed small mPEG-PLGA NPs for the delivery of schisantherin A in PD (Figure 3) [118]. It was demonstrated that these NPs promote the delivery of schisantherin A into the brain, improve the oral bioavailability, increase the brain uptake, and enhance the bioactivity of this drug.

Huang et al. synthesized PLGA NPs with a BBB-penetrating peptide for the co-delivery of Aβ generation inhibitor and curcumin in AD mice [119]. Overall, they found that these NPs were associated with a reduced level of Ab, ROS, TNF-a, and IL-6, and increased activity of superoxide dismutase (SOD) and synapse numbers in AD mouse brains, leading to their potential therapeutic use in AD. Chu et al. prepared TMZ-loaded PLGA NPs functionalized with anti-EPHA3 for targeting glioblastomas [120]. It was demonstrated that these NPs are significantly taken up by glioblastoma cells and also remarkably increase the apoptosis in tumor cells. Li et al. prepared lactoferrin-functionalized PEG-PLGA NPs for the delivery of shikonin and the treatment of gliomas [121]. A coating of lactoferrin was used to promote penetration through the BBB, and in vitro and in vivo experiments showed the great uptake and distribution of NPs in the brain, resulting in their effectiveness in the treatment of glioblastomas. Orunoglu et al. synthesized curcumin-loaded PLGA NPs for targeting gliomas [123]. They showed the decreased tumor size and increased survival of mice treated with these NPs. Zou et al. investigated the effectiveness of paclitaxel-loaded PLGA NPs in targeting brain tumor-associated macrophages [124]. Their results demonstrated the beneficial effects of these NPs in the treatment of gliomas. Also, it has been shown that brain- and brain tumor-penetrating disulfiram NPs were cytotoxic to glioma cells and intracranial xenografts [197]. Kou et al. prepared L-carnitine-conjugated PLGA NPs for targeting glioma cells [125]. These NPs were found to significantly cross the BBB and showed great antiglioma efficacy. Chai et al. synthesized efficient functionalized cell membrane-coated NPs with neurotoxin-derived peptides for brain drug delivery [198].

#### 4.1.4. Poly (ε-caprolactone) (PCL)

Poly (ε-caprolactone) (PCL) is a biodegradable polyester that is widely applied to promote toughness and enhance the flexibility of various materials such as PLA. Moreover, PCL, as a common polymer for electrospinning, has been used extensively for cell culture scaffolds [199,200,201]. PCL degrades slower than PLA and therefore is suitable to fabricate scaffolds and NPs for the long-term and sustained release of pharmaceutical agents [202,203]. Changyong et al. [126] used Poly (N-isopropyl acrylamide)-b-poly(3-caprolactone) (PNPCL) block copolymers as a thermosensitive nanosystem for the delivery of clonazepam into the brain. Results showed that Poly (N-isopropyl acrylamide) as part of a copolymer covers the surface of NPs in a layer and prevents the fast release of the drug. The role of clonazepam in the brain is the enhancement of the effects of GABA; it thereby decreases or stops specific signaling in the nerves. Rezaie et al. investigated the effect of hyperthermia and ionizing radiation on the cytotoxicity induced by IUdR-loaded PCL-PEG-coated magnetic NPs on a U87MG glioblastoma cell line [127]. It was found that these NPs have more toxicity against glioblastoma cells compared to IUdR alone. Irani et al. investigated the efficacy of CS/TMZ NPs-loaded PCL-PU nanofibers against U-87 MG human glioblastoma cells [128]. These nanofibers released TMZ for 30 days and significantly decreased the cell viability and survival of glioblastoma cells. Küçüktürkmen et al. examined the effects of pemetrexed- and miR-21 antisense oligonucleotide-loaded lipid–polymer hybrid NPs on glioblastoma cells [129]. These NPs gradually released pemetrexed over 10 h and the encapsulation of pemetrexed in lipid NPs increased the cellular uptake from 6% to 78%. Also, confocal microscopy demonstrated that anti-miR-21 enhances the accumulation of lipid NPs in the nucleus of U87MG cells. Finally, it was shown that higher cytotoxicity was achieved by the delivery of anti-miR-21 and pemetrexed through a lipid–polymer hybrid NPs. Ahmad et al. investigated the beneficial effects of eugenol-encapsulated–CS-coated-PCL NPs for the treatment of cerebral ischemia [130]. It was shown that IN administration of these NPs increases their bioavailability in the rat brain and these NPs may lead to the treatment of cerebral ischemia. Irani et al. investigated the prolonged delivery of TMZ from electrospun PCL-Diol-b-PU/gold nanocomposite nanofibers for the treatment of glioblastoma tumors [131]. Their results demonstrated the decreased viability and survival of glioblastoma cells treated with these NPs. Varan and Bilensoy synthesized cationic PEGylated polycaprolactone NPs containing post-operation docetaxel for glioma treatment [132]. Their results revealed the higher cytotoxicity of these NPs compared to docetaxel alone.

#### 4.1.5. PACA

Couvreur and co-workers were among the first to study PACA NPs in the treatment of brain diseases [204]. PACA NPs have improved some properties of drugs from the clinical point of view. Its NPs decrease the drug dosage and reduce the side effects of the drug. Also, PACA NPs improve drug bioavailability and half-life, and have a noninvasive route of administration. The most promising advantage of PACA NPs is the ability to overcome multidrug resistance (MDR) [205,206]. Cancerous cells possessing MDR are able to evade chemotherapeutic agents. Overexpressed p-glycoprotein is the main reason for MDR resistance in tumor cells [207,208,209]. Many drugs have been used for the treatment of brain diseases based on polysorbate 80-coated PACA NPs such as DOX, methotrexate, loperamide, tubocurarine, dalargin, kyotorphin, and the NMDA receptor antagonist MRZ 2/576 [210,211,212,213,214,215]. Wilson et al. [133] used PBCA NPs to deliver the anti-Alzheimer’s drug tacrine. The results showed an increase in drug concentration in the liver and the spleen. The authors modified the surface of NPs with P80 and observed the penetration of drug-loaded NPs through the BBB. In the same study, the authors used rivastigmine, a reverse cholinesterase inhibitor. They encapsulated the drug in P80-coated PBCA NPs. They observed a 4-fold increase in drug concentration in the brain, in comparison with the control [79]. Ambruosi et al. [216] studied the effect of DOX-loaded PBCA NPs coated with different kinds of surfactants (P80, P188, and poloxamine 908) on glioblastoma in a rat model. The results showed a decrease in RES uptake and an increase in the antitumoral effect of DOX-loaded PBCA NPs coated with PS80 after IV injection [217]. Petri et al. [134] investigated the possible mechanisms for the delivery of DOX-loaded PBCA NPs that were coated with different surfactants (P188 and P80). DOX-PBCA NPs coated with P188, like DOX-PBCA NPs coated with P80, considerably increased the median survival rates and antitumor effect of DOX against an intracranial glioblastoma in rats. The authors found that ApoA-I adsorb considerably on the surface of NPs. The results showed that DOX-loaded PBCA NPs stabilized by P188 also produced considerable antitumor effects. Endocytosis through the endothelial cells of the brain capillary is the main strategy NPs use to cross the BBB [138]. Some of the plasma proteins adsorb, especially on the surface of P80-coated PBCA NPs, and trigger the receptor-mediated endocytosis of NPs and finally their penetration into the brain [218]. Yamamoto et al. [219] used PBCA NPs for the entrapment of TMZ. TMZ is a DNA-methylating agent applied for the treatment of melanoma and brain tumors. PBCA NPs showed controlled release of TMZ. The authors suggested that TMZ-PBCA NPs can be used for TMZ delivery to the brain without any effect on drug hydrolysis. Wang et al. [220] prepared P80-coated gemcitabine–PBCA NPs (GCTB–PBCA-NPs). GCTB is a pyrimidine nucleoside analog anticancer agent that has shown promising antitumor activity. C6 glioma cells treated with these NPs demonstrated the most dramatic changes in growth status and cell morphology. GCTB-PBCA NPs inhibited cell growth by arresting G0/G1 to S phase transition, and cell proliferation slowed down significantly. Also, dramatic changes in cell morphology were observed, including nuclear vacuoles, ruptured cells, and dead cells and cell debris in the medium. Mulik et al. [135] used Apo E3–PBCA NPs as a curcumin delivery system to inhibit Aβ and related oxidative stress in AD. Apo E3–PBCA NPs increased curcumin stability and sustained release. The results showed that the anti-apoptotic activity of Apo E3- curcumin-PBCA was increased 2–3-fold in comparison with curcumin–PBCA NPs. The authors suggested that ApoE3–curcumin–PBCA NPs can significantly increase the uptake of a drug across the BBB. Kurakhmaeva et al. [136] studied the antiparkinsonian effect of NGF–PBCA NPs coated with P80 in a mouse model for PD. The results showed improvement of the symptoms of oligokinesia after the administration of P80-(NGF)–PBCA NPs. Kreuter et al. [137] investigated the effect of different surfactants (poloxamer 184, 188, 388, 407 and poloxamine 908; polysorbate 20, 40, 60, 80) on dalargin delivery across the BBB by PBCA NPs. The results showed that after IV injection of surfactant-dalargin-PBCA NPs to mice, only the surfactants polysorbate 20, 40, 60, and 80 were able to induce the passage of dalargin across the BBB. The authors showed that P80 enabled the highest induction of analgesia at both dosages of dalargin, 7.5 mg/kg as well as 10 mg/kg. Kreuter et al. [138] confirmed that P80–PBCA NPs can deliver significant amounts of dalargin to the CNS. PEGylation of PACA NPs is another technology that can be used in drug delivery to the brain [15,77,178,221,222,223,224,225]. These PEGylated NPs are able to cross the BBB and penetrate the brain. It has been shown, however, that PACA NPs have limitations in clinical applications. The by-products that result from PBCA degradation can stimulate or damage the CNS [226]. In addition, it has been demonstrated that this kind of nanocarrier is not a good candidate for the treatment of chronic diseases because of the short duration of the pharmacological effect [80,227].

### 4.2. Natural Polymers

#### 4.2.1. HSA

**Figure 4:**
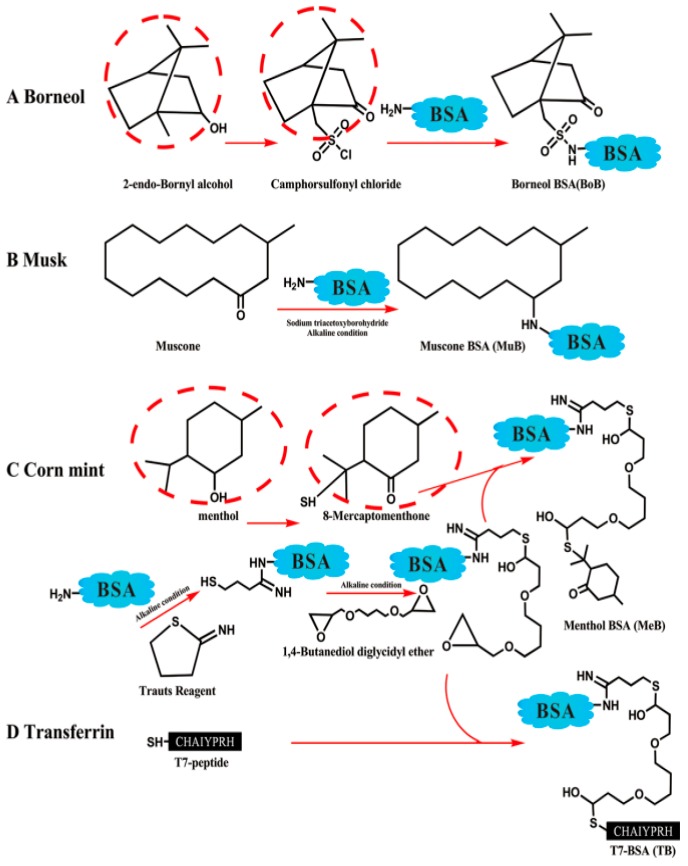
Schemes of modified albumin including borneol BSA (**A**), muscone BSA (**B**), menthol BSA (**C**), and T7 BSA (**D**). Reproduced with permission from Liang, J.; Gao, C.; Zhu, Y.; Ling, C.; Wang, Q.; Huang, Y.; Qin, J.; Wang, J.; Lu, W.; Wang, J. Natural Brain Penetration Enhancer-Modified Albumin Nanoparticles for Glioma Targeting Delivery. *ACS Appl. Mater. Interfaces* 2018 [147].

**Figure 5:**
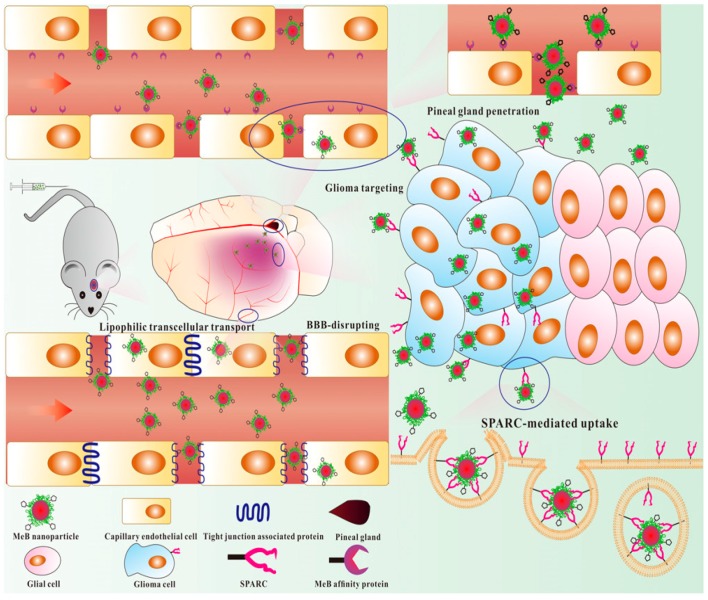
Schematic diagram of possible BBB penetration pathways and glioma-targeting ability of BPE–BSA-based NPs. Reproduced with permission from Liang, J.; Gao, C.; Zhu, Y.; Ling, C.; Wang, Q.; Huang, Y.; Qin, J.; Wang, J.; Lu, W.; Wang, J. Natural Brain Penetration Enhancer-Modified Albumin Nanoparticles for Glioma Targeting Delivery. *ACS Appl. Mater. Interfaces* 2018 [147].

HSA is a water-soluble and low-molecular-weight (~66 kDa) protein. Some of the major roles of this abundant protein in the body include: enhancing the solubility of long-chain fatty acids, the transportation of different ions and compounds such as drugs and hormones, and the regulation of osmotic pressure in the blood circulation system [228]. These desirable characteristics, in addition to the long half-life of approximately 20 days in the circulation system, make it a potential candidate for drug delivery. NPs made of HSA have been investigated and fabricated for drug delivery to the brain and their applications in diagnostics and therapeutics [229,230]. Ulbrich et al. [92] investigated a stable nanoparticulate system to transport loperamide across the BBB. The authors used HSA NPs with covalently bound Tf or TfR mAbs (OX26 or R 17217). The results of the tail-flick test demonstrated that targeted HSA NPs were able to deliver loperamide across the BBB and induce antinociceptive (analgesic) effects in the brain. In another study, the same authors used HSA NPs with covalently attached insulin or an anti-insulin receptor monoclonal antibody to deliver loperamide across the BBB. The results showed that NPs induced significant antinociceptive effects in the tail-flick test [144]. Dadparvar et al. [231] investigated the binding of HI 6 dimethanesulfonate and HI 6 dichloride monohydrate to HSA NPs for the treatment of poisoning by organophosphorus compounds. In vitro assessment of the drug activity showed that HSA NPs transport drugs through the blood–brain barrier. Zensi et al. [229] prepared HSA NPs targeted with covalently bound Apo E by a desolvation technique. The authors intravenously injected Apo E-HSA NPs into the SV 129 mice. Apo E-HSA NPs were detected in the brain and neurons of SV 129 mice after 15 and 30 min. They suggested that drug-loaded NPs’ entrance into the brain requires an interaction between Apo E and the LDL receptor family and receptor-mediated endocytosis. Similar results were observed in other studies [232,233]. Ruan et al. synthesized substance P-modified HSA NPs containing paclitaxel for targeting gliomas [145]. These NPs had great properties in terms of drug-loading content (7.89%), entrapment efficiency (85.7%), spherical structure with a size of 150 nm, and zeta potential of –12.0 mV. These NPs showed great uptake by brain capillary endothelial cells and U87 cells; in addition, it was found that they were toxic for glioma cells, so they can be considered as novel agents for targeting gliomas. Wong and Ho investigated the efficiency of serum albumin as a nanoparticulate carrier for the delivery of R-flurbiprofen and the treatment of AD [146]. Their results showed that the IN route to administration of these NPs is preferred to oral and IN administration of a simple R-flurbiprofen solution so that a higher brain to-plasma ratio profile was achieved by this administration and it was demonstrated that these NPs are potential therapeutic agents for the amelioration of mitochondrial dysfunction in AD. Liang et al. prepared carriers by conjugation of borneol, muscone, and menthol to BSA for targeting gliomas (Figure 4) [147]. BSA was found to improve the drug accumulation in the glioma region after penetration of the blood–brain barrier (BBB) (Figure 5).

These carriers had great biocompatibility; it was found that they efficiently penetrate the BBB and are captured by cells, showing their efficiency for drug delivery in gliomas.

#### 4.2.2. Gelatin (GE)

Gelatin (GE) is a by-product of denatured and partially hydrolyzed collagen, which is extensively used in tissue engineering and therapeutic delivery [234,235]. Also, GE has bioactive materials such as arginine–glycine–aspartic acid, which gives GE a cell attachment property and makes GE valuable as a biomaterial [28,236,237,238,239,240,241]. At present, GE and its blends are used in the food industry and in medical products [242,243]. The nontoxic, biodegradable, and bioactive properties of GE make it a suitable carrier for drug delivery [244]. Kaur et al. [148] encapsulated an anti-HIV drug (hydrophilic didanosine) in GE NPs coated with mannan. Free didanosine drug is not able to penetrate the BBB. However, the brain concentration of the drug increased after the administration of GE NPs. GE–siloxane NPs improved the delivery of a model drug (rhodamine B isothiocyanate) to the brain. A cell-penetrating peptide (SynB) was conjugated to the NPs. The nanocomplexes were efficiently taken up by brain capillary endothelial cells and showed proper biocompatibility and nontoxicity [149]. Nejat et al. synthesized cardamom extract-loaded GE NPs for the treatment of glioblastomas [150]. These NPs had a diameter in the range of 40–200 nm, a zeta potential of −40.1 mV, and entrapment efficiency of 70%, and it was found that these NPs have cytotoxic effects on human glioblastoma cancer U87MG cells. 

#### 4.2.3. Chitosan (CS)

CS is a water-soluble cationic polysaccharide with a positive charge and biocompatible and biodegradable characteristics [245,246]. The nonallergenic and nontoxic characteristics of CS make it an appropriate choice for delivery systems in pharmaceutical applications [247,248,249]. CS has the ability to make epithelial cells permeable by means of its interaction with the tight junction and then opening them to cross the epithelial barrier [250]. The absorption and penetration of different kinds of drugs (e.g., proteins, peptides, hormones, etc.) have been studied through the nasal epithelium [251,252,253]. IN administration of interferon β-1b (IFNβ-1b) was investigated by Ross et al. [254] in multiple sclerosis. The aim was the targeted delivery of IFNβ-1b to the rat CNS. Autoradiography studies showed the active and efficient delivery of IFNβ-1b to the CNS by IN administration. Delivery of IFNβ-1b to the monkey CNS has been studied by Thorne et al. [255]. This work was the first investigation to determine CNS drug distribution in nonhuman primates. Studies showed that IN administration of IFNβ-1b resulted in inefficient targeting of the CNS. The authors concluded that IN administration of drugs is a noninvasive method to access the brain and direct drugs to the CNS. Moreover, new technology-based delivery systems have been used for drug delivery to the brain through the nose to brain pathway or IN administration. Wang et al. [151] fabricated estradiol-loaded CS NPs by the ionic gelation method. The authors compared the uptake of the drug into the cerebrospinal fluid (CSF) between the two methods of administration (IN and IV). The results confirmed the efficient delivery of estradiol to the CSF by CS NPs through the IN route of administration. Aktas et al. [152] investigated the brain delivery of anticaspase peptide Z-DEVD-FMK by CS-poly(ethylene glycol) (PEG). They used the avidin–biotin system to attach the OX26 monoclonal antibody to the surface of NPs. The results showed that, after the intravascular administration of CS–PEG–biotin–avidin/OX26, NPs can lead to the delivery of anticaspase peptide Z-DEVD-FMK to the brain, outside of the intravascular compartment. Trapani et al. [154] prepared and characterized dopamine-loaded CS NPs. In vivo results showed enhanced brain delivery of dopamine by these nanocarriers. Moreover, CS NPs have shown their potential in the development of a delivery system to overcome the BBB problem [256]. Tammam et al. synthesized CS NPs for nuclear and cytoplasmic delivery of lactoferrin in gliomas [155]. Interestingly, it was found that the cytotoxicity of NPs containing lactoferrin on gliomas is due to their cytoplasmic allocation. Gu et al. prepared antibody-modified CS NPs to deliver siRNA for targeting HIV replication in astrocytes [156]. It was demonstrated that the antibody moiety increases the knockdown effect of siRNA-loaded NPs, showing their efficiency in inhibiting HIV replication in astrocytes. Gholami et al. investigated the proficiency of super-paramagnetic iron oxide/DOX-loaded CS NPs for glioblastoma theranostics [157]. Magnetic resonance imaging (MRI) demonstrated the high uptake of NPs by C6 glioma cells, showing their application in the diagnosis of glioblastoma. Xu et al. investigated the effect of lactoferrin-coated polysaccharide NPs based on CS hydrochloride/hyaluronic acid/PEG on brain gliomas [158]. These NPs were remarkably captured by brain capillary endothelial cells and can penetrate the BBB. Also, 1, 3β-glucan-anchored paclitaxel-loaded CS cross-linked targeted NPs have been proposed as potential therapeutic options for the treatment of brain tumors [159,160].

## 5. Conclusions

Although the BBB acts as vital protection for the brain against foreign substances, its structure is a major obstacle to the delivery of drugs into the brain for the treatment of CNS diseases. Most of the drugs that are presently being used for brain diseases use the IV route of administration and thereby face the reticuloendothelial system (RES), which removes them from the bloodstream. Also, it has been shown that this route of drug administration is related to systemic distribution and side effects and reduces the drug efficacy and bioavailability. Progress in nanotechnology-based drug delivery systems has overcome some but not all of these problems. Conditional and targeted polymeric NPs that are sensitive to the specific situation or environment for controlled and sustainable drug release, in combination with the IN route of administration, are the newest technology for drug delivery into the brain. This targeted delivery via IN administration solves the major problems involving the BBB, increases drug efficacy, and decreases drug side effects.

## Figures and Tables

**Table 1:** Some selected polymeric NPs for the delivery of drugs to the brain.

Polymers	Model Drug/Other Molecules	Remarks	References
PLA	Neurotoxin-I NT-I	Intranasal (IN) administration of NT-I-PLA is more effective than IV administration	[103]
Polylactic acid-co- hyperbranched polyglycerol modified with adenosine (PLA–HPG–Ad)	Camptothecin (CPT)	Increased BBB uptake after IV injection	[104]
PLA	Thyrotropin-releasing hormone (TRH)	Effective IN administration	[105]
PLA–TPGS	Tf	More effective compared to NPs without Tf	[106]
PLA–PEG–CPPs	NT-I	High concentration of drug in the brain through IN administration	[107]
PLA	Neuropeptide	High drug delivery in IN administration	[108]
PLG–PEG–H102	H102 peptide (HKQLPFFEED)	High uptake and biocompatibility, and high concentration of coumarin-6 in the brain following IV administration	[109]
PLA–mPEG–Lf	Tf	Reduced toxicity	[110]
PLA–MPS–LDLR	Resveratrol	Increased the migration of NPs through the BBB	[111]
PLGA–CS, P80, and P188	-	Prolonged circulation in the blood, high cellular uptake	[112]
PLGA–PVA or HSA/P80 or P188	Doxorubicin (DOX) and loperamide	Crossed the BBB and released the drug at a specific site	[113]
Tween 80–PLGA	Estradiol	High level of the drug in the brain after oral administration	[114]
Trimethylated chitosan (TMC)–PLGA	Coenzyme Q10	Low toxicity and good penetration into the brain matrix	[115]
PLGA–PEG	Pep TGN	High accumulation of NPs in the brain after IV injection	[116]
PEGylated-PLGA	Memantine	Decreased amyloid-beta (Aβ) plaques and related inflammation characteristics	[117]
mPEG–PLGA	Schisantherin A	Improved oral bioavailability, increased brain uptake, and enhanced the bioactivity of this drug	[118]
Rabies virus glycoprotein 29-modified deferoxamine-loaded PLGA	Deferoxamine	Significantly decreased dopaminergic neuron damage	[26]
BBB-penetrating peptide–PLGA	Aβ generation inhibitor and curcumin	Increased activity of superoxide dismutase (SOD) and synapse numbers in the AD mouse brains	[119]
PLGA–anti-EPHA3	Temozolomide (TMZ)	Significantly taken up by glioblastoma cells, remarkably increased apoptosis	[120]
Lf-PLGA–PEG	Shikonin, lactoferrin	Great uptake and distribution in the brain	[121]
PLGA	Ropinirole (RP)	Reverted PD-like symptoms of neurodegeneration in the animal model	[122]
PLGA	Curcumin	Decreased tumor size and increased survival of animal model	[123]
PLGA	Paclitaxel	Increased treatment of glioma	[124]
PLGA	L-carnitine	Significantly crossed the BBB, great antiglioma efficacy	[125]
Poly(N-isopropyl acrylamide)-b-poly(caprolactone) (PN-co-PCL)	Clonazepam	Prevented fast release of the drug	[126]
PCL-PEG	5-iodo 2′ deoxyuridine (IUdR)	High toxicity against glioblastoma cells	[127]
Poly (ε-caprolactone diol)-based polyurethane (PCL-Diol-b-PU)	CS and TMZ	Significantly decreased the cell viability and survival of glioblastoma cells	[128]
Lipid polymer nanoparticles (LPN)	Pemetrexed- and miR-21 antisense oligonucleotide	Increased the cellular uptake and gradually released of pemetrexed	[129]
PCL	Eugenol encapsulated CS	Increased bioavailability for the treatment of cerebral ischemia	[130]
PCL-Diol-b-PU/gold	TMZ	Decreased viability and survival of glioblastoma cells	[131]
PEGylated PCL	Docetaxel	Effective cytotoxicity	[132]
Polysorbate 80-coated PBCA	Tarcrine	Increased drug concentration in the brain	[133]
PBCA–P80 or P188	DOX	Considerable antitumor effects	[134]
PBCA–Apo E3	Curcumin	Increased antiapoptotic activity of Apo E3- curcumin-PBCA NPs	[135]
PBCA–P80	Nerve growth factor (NGF)	Moderation in symptoms of oligokinesia	[136]
PBCA–P20,40,60,80,184,188,388,407, and 908	Dalargin	The surfactant polysorbate 80 enabled the highest induction of analgesia at both dosages of dalargin	[137]
PBCA-P80	Dalargin	Efficient delivery of drugs into the brain	[138]
P(HDCA-co-RCA-co-MePEGCA) and ^14^C-P(HDCA-co-MePEGCA)	Anti-Aβ1-42	Completed correction of the memory defect in an experimental model of AD	[139]
PLGA functionalized with OX26-type monoclonal antibody	TMZ	Cytotoxicity improvement of TMZ	[140]
PLGA–b-PEG-ascorbic acid	Galantamine (GLM)	High biodistribution, therapeutic, and sustained action of the drug	[141]
1,2-distearoyl-sn-glycero-3- phosphoethanolamine-N-[amino(polyethylene glycol)-2000 (DSPE-PEG2000)	Poly(benzodithiophene-alt-benzobisthiadiazole)	Efficient near-infrared (NIR) II PA imaging of orthotropic brain tumor	[142]
Polyacrylamide (PAAM)-cardiolipin (CL)- PLGA grafted with 83-14 monoclonal antibody (MAb)	Curcumin (CUR) and Rosmarinic acid (RA)	Permeated the BBB and reduced the fibrillar Aβ-induced neurotoxicity	[143]
HSA–Tf or TfR mAbs	Loperamide	Loperamide delivery across the BBB induced antinociceptive (analgesic) effects	[92]
HSA–insulin or anti-insulin receptor monoclonal antibody	Loperamide	Induced significant antinociceptive effects in the tail-flick test	[144]
HSA	Paclitaxel	Great uptake by brain capillary endothelial cells and U87 cells	[145]
Serum albumin	R-flurbiprofen	Higher brain to-plasma ratio profile, amelioration of mitochondrial dysfunction in AD	[146]
BSA	Borneol, muscone, and menthol	The biocompatible carriers efficiently penetrate the BBB and are captured by cells	[147]
Gelatin (GE)–mannan	Anti-HIV drug (hydrophilic didanosine)	Increased brain concentration of the drug	[148]
GE–siloxane–SynB	rhodamine B isothiocyanate	The biocompatible nanocomplexes were efficiently taken up by brain capillary endothelial cells	[149]
GE–cardamom extract		Cytotoxic effects on U87MG cells	[150]
CS	Estradiol	Efficient delivery of estradiol to the cerebrospinal fluid (CSF) through IN administration	[151]
CS–PEG–biotin–avidin/OX26	Anticaspase peptide Z-DEVD-FMK	Z-DEVD-FMK delivery to the brain, outside of the intravascular compartment	[152]
CS–β-cyclodextrin	Estradiol	Significantly increased the amount of estradiol in the CSF	[153]
CS	Dopamine	Enhanced brain delivery of dopamine	[154]
CS	Lactoferrin	Cytoplasmic allocation of the NPs	[155]
Antibody-modified CS	siRNA	Showing their efficiency in inhibiting HIV replication in astrocytes	[156]
CS–iron oxide	DOX	High uptake of NPs by C6 glioma cells, showing their application in the diagnosis of glioblastoma	[157]
CS hydrochloride/hyaluronic acid/PEG	Lactoferrin	The NPs can penetrate the BBB	[158]
CS-1, 3-glucan	Paclitaxel	Potential therapeutic options are demonstrated	[159,160]
CS	Pramipexole dihydrochloride (P)	Superior in vivo activity for brain targeted delivery in Parkinson’s disease	[161]
CS-based hydrogel	Methotrexate (MTX)	Facilitated MTX passage by providing a higher concentration of the drug in contact with the BBB	[162]

## Abbreviations

AD	Alzheimer’s disease
PD	Parkinson’s disease
PLA	Polylactic acid
PGA	Polyglycolic acid
PLGA	Poly D,L-lactide-co-glycolide
PVA	Polyvinyl alcohol
PCL	Poly (ε-caprolactone)
PNPCL	Poly (*N*-isopropylacrylamide)-b-poly(3-caprolactone)
PACA	Poly (alkyl cyanoacrylate)
PBCA	Poly (butyl cyanoacrylate)
PEG	Polyethylene glycol
P80	Polysorbate 80
P188	Poloxamer 188
HSA	Human serum albumin
CS	Chitosan
CPP	Cell-penetrating peptides
BBB	Blood–brain barrier
CNS	Central nervous system
GE	Gelatin
NDDs	Neurodegenerative diseases
MS	Multiple sclerosis
NPs	Nanoparticles
RES	Reticuloendothelial system
ROS	Reactive oxygen species
SOD	Superoxide dismutase
CSF	Cerebrospinal fluid
IV	Intravenous
IN	Intranasal
MDR	Multidrug resistance
NT-1	Neurotoxin-I
TRH	Thyrotropin-releasing hormone
Lf	Lactoferrin
LDLR	Low-density lipoprotein receptor
DOX	Doxorubicin
Aβ	Amyloid beta
GABA	Gamma-aminobutyric acid
TMZ	Temozolomide
Tf	Transferrin
TMC	Trimethylated chitosan (TMC)
GCTB	Gemcitabine
NGF	Nerve growth factor
Apo	Apolipoprotein
IFNβ-1b	Interferon β-1b
